# Ventricular dyssynchrony late after the Fontan operation is associated with decreased survival

**DOI:** 10.1186/s12968-023-00984-3

**Published:** 2023-11-20

**Authors:** Addison Gearhart, Sunakshi Bassi, Rahul H. Rathod, Rebecca S. Beroukhim, Stuart Lipsitz, Maxwell P. Gold, David M. Harrild, Audrey Dionne, Sunil J. Ghelani

**Affiliations:** 1 Department of Cardiology, Boston Children’s Hospital, 300 Longwood Ave, Boston, MA 02115 USA; 2 Department of Pediatrics, Harvard Medical School, Boston, MA USA; 3 Division of General Internal Medicine, Brigham and Women’s Hospital, Boston, MA USA; 4 Massachusetts Institute of Technology, Boston, MA USA

**Keywords:** Congenital heart disease, Dyssynchrony, Fontan, Cardiac magnetic resonance imaging

## Abstract

**Background:**

Ventricular dyssynchrony and its relationship to clinical outcomes is not well characterized in patients following Fontan palliation.

**Methods:**

Single-center retrospective analysis of cardiac magnetic resonance (CMR) imaging of patients with a Fontan circulation and an age-matched healthy comparison cohort as controls. Feature tracking was performed on all slices of a ventricular short-axis cine stack. Circumferential and radial strain, strain rate, and displacement were measured; and multiple dyssynchrony metrics were calculated based on timing of these measurements (including standard deviation of time-to-peak, maximum opposing wall delay, and maximum base-to-apex delay). Primary endpoint was a composite measure including time to death, heart transplant or heart transplant listing (D/HTx).

**Results:**

A total of 503 cases (15 y; IQR 10, 21) and 42 controls (16 y; IQR 11, 20) were analyzed. Compared to controls, Fontan patients had increased dyssynchrony metrics, longer QRS duration, larger ventricular volumes, and worse systolic function. Dyssynchrony metrics were higher in patients with right ventricular (RV) or mixed morphology compared to those with LV morphology. At median follow-up of 4.3 years, 11% had D/HTx. Multiple risk factors for D/HTx were identified, including RV morphology, ventricular dilation, dysfunction, QRS prolongation, and dyssynchrony. Ventricular dilation and RV morphology were independently associated with D/HTx.

**Conclusions:**

Compared to control LVs, single right and mixed morphology ventricles in the Fontan circulation exhibit a higher degree of mechanical dyssynchrony as evaluated by CMR-FT. Dyssynchrony indices correlate with ventricular size and function and are associated with death or need for heart transplantation. These data add to the growing understanding regarding factors that can be used to risk-stratify patients with the Fontan circulation.

**Supplementary Information:**

The online version contains supplementary material available at 10.1186/s12968-023-00984-3.

## Introduction

Despite significant improvements in life expectancy after the Fontan operation, patients with functional single ventricles often experience progressive heart failure and increased risk of premature death or heart transplantation (D/HTx) [1–3]. The estimated 20-year survival for patients palliated to a Fontan circulation is 61–85% with a risk of late mortality of approximately 2% per year [4].

Synchrony plays an important role in efficient ventricular pump function and cardiac output. In a normal 2-ventricle circulation, the fast activation of electrical conduction through the heart is reflected by a narrow QRS on electrocardiogram (ECG) and a synchronous pattern of contraction. Left ventricular dyssynchrony is defined by temporal differences in activation (electrical dyssynchrony) and contraction (mechanical dyssynchrony) of various myocardial segments of the left ventricle. In adult and pediatric patient populations with biventricular circulations, ventricular dyssynchrony has been associated with life-threatening arrhythmias [5], heart failure [6, 7], and mortality [8–10]. Feature tracking (FT) to derive strain parameters has been proposed as a sensitive tool to evaluate global and regional myocardial deformation and mechanical ventricular dyssynchrony in patients with functional single ventricles [11]. Prior echocardiography-based studies in children with functional single ventricles have demonstrated high rates of ventricular dyssynchrony, particularly for patients with hypoplastic left heart syndrome [12–14]. In patients with a Fontan circulation mechanical dyssynchrony has been associated with reduced ejection fraction (EF), longer QRS duration, and composite outcomes such as unplanned hospitalizations and/or death [15]. However, prior studies have not provided a detailed characterization of patient-related factors (e.g. underlying ventricular morphology) that are associated with dyssynchrony, and their relative contribution to patient outcomes. Moreover, prior studies have been limited by smaller sample sizes and relative paucity of outcomes such death or need for heart transplantation.

The present study uses cardiac magnetic resonance (CMR) to characterize mechanical dyssynchrony in functional single ventricles using FT. It describes novel dyssynchrony metrics and analyzes their relationship with ventricular morphology, volumetric and function data, and QRS duration. In addition, the study evaluates the association between dyssynchrony and clinical outcomes including death and need for heart transplantation.

## Methods

This was a single-center, retrospective cohort study. The Institutional Review Board approved the study and waived the need for informed consent.

### Study population

**Fig. 1:**
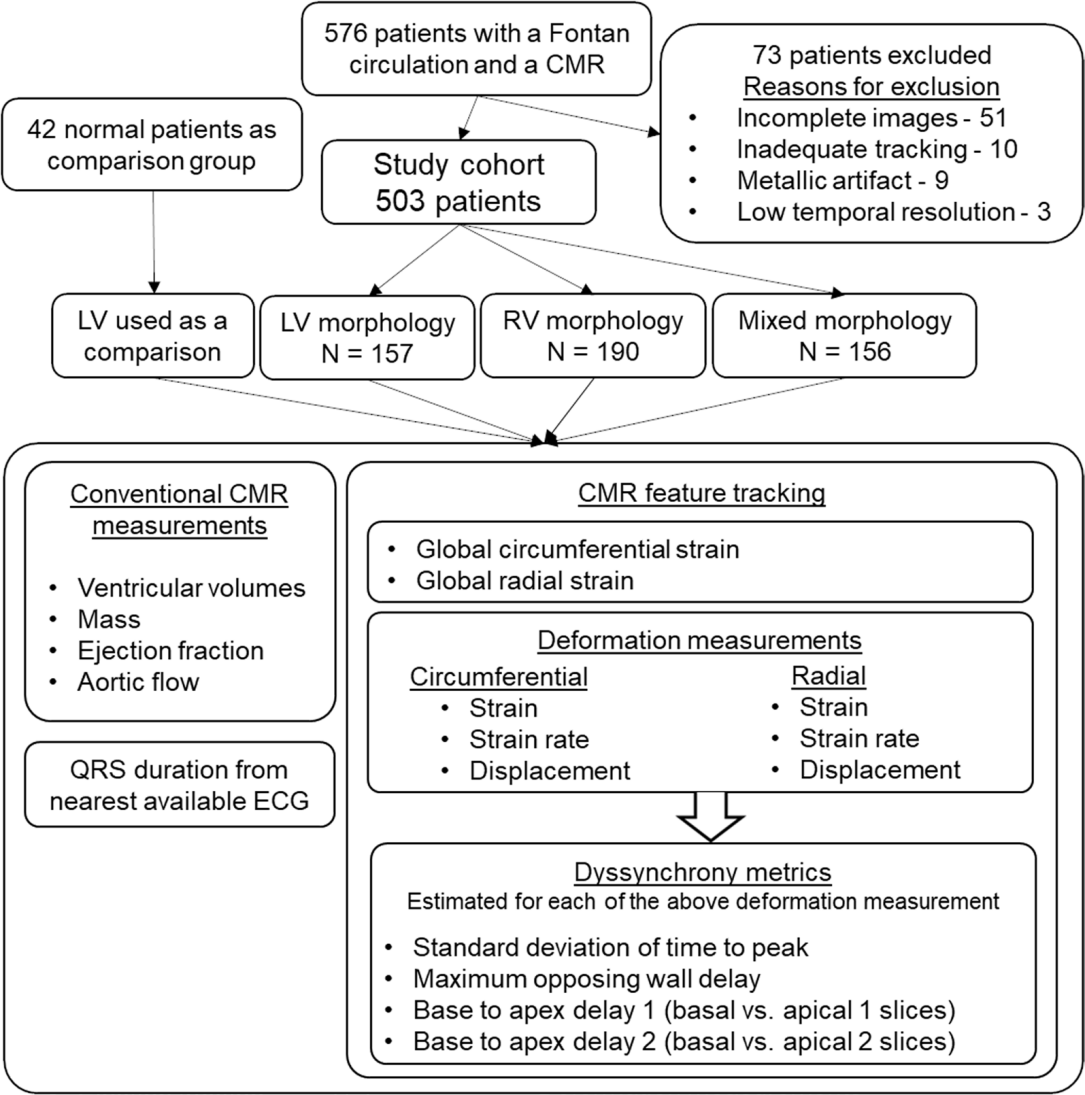
Study workflow. *CMR* cardiac magnetic resonance, *LV* left ventricle, *RV* right ventricle, *ECG* electrocardiogram

All patients with a Fontan circulation who had at least one available CMR after 7/1/05 through 9/23/21 were screened for eligibility. The study workflow is illustrated in Fig. 1. Patients with a pacemaker were excluded as it is a relative contraindication to CMR and rarely undergo CMR examinations at our center. Ventricular morphology was designated as either LV or RV, as appropriate, if the non-dominant ventricle was ≤ 20% of the combined end diastolic volume (EDV) and mixed type if the non-dominant ventricle was > 20% of the combined EDV [16]. When multiple studies were available per patient, the earliest CMR was analyzed. Patients with inadequate image quality for FT analysis were excluded. In the instance of poor image quality, a subsequent CMR was used when available. A comparison group was identified as individuals referred for CMR for suspected cardiomyopathy or congenital heart disease (CHD) but whose studies were subsequently interpreted as normal and at the time of study acquisition or interim follow-up did not have known systemic or genetic disease. The comparison group was age-matched ~ 1:12 using age in years in a random order due to the limited availability of normal studies. Demographic and clinical data were extracted from electronic medical records.

### CMR examinations

Fontan imaging was conducted according to standard practice at our center, as has been previously described [17]. Briefly, studies were performed on a 1.5T scanner (Achieva, Philips Healthcare, Best, the Netherlands) using surface coils appropriate for patient size. A ventricular short-axis balanced steady-state free precession (bSSFP) cine stack with breath-holding and ECG-gating was used for volumetric and FT analysis. Typical slice thickness was 8–10 mm, spatial resolution was 1.7–2 × 1.7–2 mm and temporal resolution was 30–40 ms with 30 reconstructed phases per cardiac cycle. Ventricular volumes and blood flow were measured using commercially available software (cvi42, Circle Cardiovascular Imaging Inc., Calgary, Alberta, Canada; and QMass, Medis Medical Imaging Systems, Leiden, the Netherlands). The following conventional measurements were recorded: indexed end-diastolic volume (EDV_*i*_), indexed end-systolic volume (ESV_*i*_), indexed stroke volume (SV_*i*_), EF, indexed ventricular mass (Mass_i_), and ascending aortic flow as a measure of cardiac output. When two ventricles contributed to the systemic circulation (i.e. mixed type ventricles), their mass and volumes were combined. For the comparison cohort, only the LV measurements were considered.

### Feature tracking and dyssynchrony indices

Feature tracking analysis was performed on the short-axis cine stack of images as previously described [18]. Briefly, the endocardial and epicardial borders were manually traced at end-diastole for all slices from the apex to base of the dominant ventricle in the case of a single LV or single RV. For mixed-type ventricles, borders were traced around the free wall of both ventricles (excluding the ventricular septum). A commercial feature tracking program was used for the analysis (cvi42, Circle Cardiovascular Imaging Inc., Calgary, Alberta, Canada). Adequacy of feature tracking was assessed by visual inspection and when not satisfactory, attempts were made to improve tracking by adjusting the baseline contour. If significant time elapsed (typically 5 min) without achieving reliable tracking, the case was excluded. The apical slice was defined as the most apical slice with blood pool through the entire cardiac cycle. The basal slice was defined as the most basal slice with a full rim of myocardium through the entire cardiac cycle. A minimum of 4 slices was required. Studies with artifact at the base of the heart that precluded accurate volumetric analysis were included if the most basal slice with circumferential myocardium was free of artifact and suitable for FT. For the comparison cohort, the same analysis was performed on the LV.

**Fig. 2:**
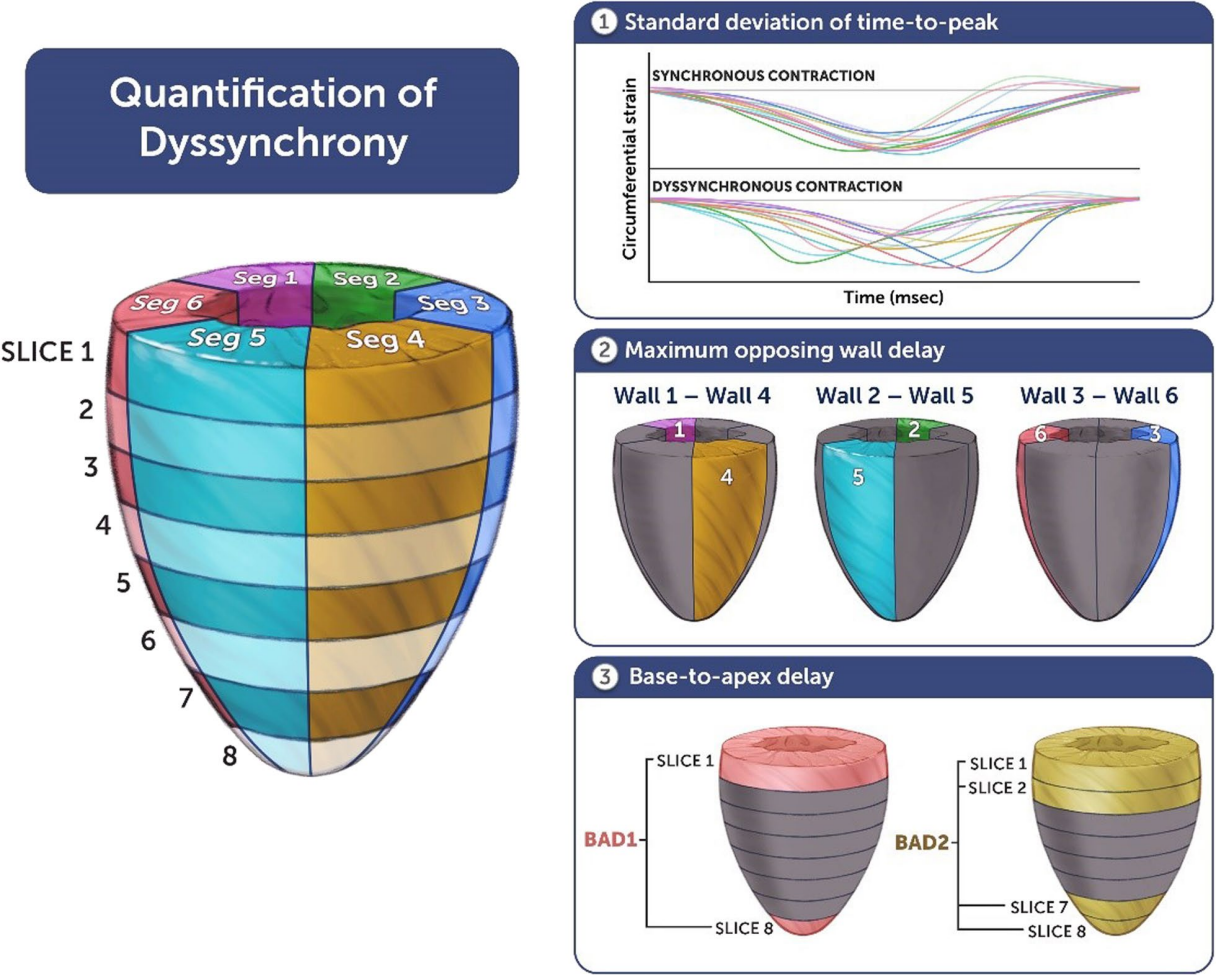
Indices of mechanical dyssynchrony. Panels 1, 2, and 3 demonstrate the various indices of dyssynchrony estimated using CMR-FT. These measures were calculated for all six indices of deformation albeit only circumferential strain is shown as an example in box 1. CMR-FT, cardiac magnetic resonance-feature tracking; BAD1, base-to-apex delay using basal and apical slices; BAD2, base-to-apex delay using basal 2 and apical 2 slices

From this, the following six deformation measurements were obtained: circumferential strain (CS), circumferential strain rate (CSR), circumferential displacement (CD), radial strain (RS), radial strain rate (RSR), radial displacement (RD). These deformation measurements were used to calculate quantify dyssynchrony using 4 methods: (1) standard deviation of time-to-peak (SDTTP) for circumferential and radial deformation measurements for all segments, (2) maximum opposing wall delay (MOWD) as the maximum difference in the average time-to-peak for 3 opposing wall pairs, and (3) base-to-apex delay (BAD) as the time difference between peak deformation (average of 6 segments) of the most basal slice and the most apical slice (BAD1) as well as (4) the difference between the peak deformation for the 2 most basal and apical slices, respectively (BAD2). As such, 24 dyssynchrony indices were derived from the FT data (Fig. 2). Global deformation measures were recorded as global circumferential strain (GCS), global circumferential strain rate (GCSR), global radial strain (GRS), and global radial strain rate (GRSR). QRS duration was captured as a marker of electric dyssynchrony from the ECG closest to the CMR from all available ECGs, with no intervening surgical or catheter-based procedure having been performed. Heart rate was recoded from the CMR report and maximal heart rate (MHR) was calculated by using the Tanaka equation (MHR = 208 − 0.7 * [age at CMR]). Heart rate was standardized as percent MHR (%MHR) by dividing the heart rate by MHR [19].

### Outcomes

The primary outcome was a time-to-event composite outcome of all-cause mortality or heart transplant listing (D/HTx). For time-to-event analysis, follow-up was measured from the date of CMR to the composite outcomes (earliest event in case of multiple) or last known documented follow-up in the medical record. If the first occurrence of transplant listing was prior to the CMR, it was excluded as an outcome.

### Statistical analysis

Data are presented as a median with interquartile ranges (IQR) as appropriate for continuous variables and as a frequency (percentage) for categorical variables. Continuous variables were compared between groups using a Mann–Whitney U test while proportions were compared using a Fisher’s exact test. Correlation between continuous variables was quantified using the Pearson’s correlation coefficient (r). Using a set of 11 a priori chosen predictors, bivariate and multivariable Cox regression analyses were performed to assess relationships between predictors and time to composite outcome (time to D/HTx, whichever comes first, or censored at time of last follow-up without these events). Approximately 7% of patients’ CMR examinations (36 out of 503) were missing data. Schafer [20] found that a rate of missing data of 5% or less likely results in minimal bias, while Bennett [21] found that a missing rate of 10% or more could lead to bias. As we were between these two rates, missing data for the predictors in the Cox regression model were addressed with multiple imputation to prevent bias [22, 23]. Based on the number of the composite outcome events, an a priori threshold of at most 5 predictors to be included in multivariable Cox model (i.e. maximum of 1 predictor for 10 outcomes) was set. Predictors chosen for the final Cox regression model were based on a forward selection Akaike Information Criterion (AIC). Kaplan Meier survival curves with log rank tests were constructed to compare freedom from the composite outcome between groups. The optimal cut point for a continuous covariate in predicting the outcomes was obtained as the value that maximizes the score statistic (equivalent to the log rank statistic for the survival outcome) [24]. Because this is an exploratory analysis, we did not adjust for multiple testing. A two-sided p-value of ≤ 0.05 was considered statistically significant. Adjustment multiple testing for Tables 1 and 2 by the Benjamini–Hochberg method did not change significance threshold level of 0.05. Statistical analyses were performed using SAS version 9.4 (SAS Institute, Cary, NC) and SPSS version 27 (IBM Corp, Armonk, NY).

**Table 1:** Baseline characteristics, conventional CMR data, and dyssynchrony measurements for all patients and comparisons

	All Fontan patients (N = 503)	Comparison group LV (N = 42)	Fontan subgroups			p-value				
			LV (N = 157)	RV (N = 190)	Mixed (N = 156)	All Fontan vs. comparison group LV	LV vs. comparison group LV	LV vs. RV	RV vs. comparison group LV	Mixed vs. comparison group LV
Baseline characteristics										
Age at CMR, y	15.2 (10.3, 21.3)	15.7 (11.0, 19.7)	16.6 (12.6, 23.8)	14.2 (10.0, 18.8)	14.8 (9.6, 21.7)	0.923	0.107	< 0.001*	0.290	0.942
Sex, male	302 (60%)	18 (43%)	93 (60%)	123 (65%)	86 (55%)	0.030*	0.049*	0.366	0.010*	0.160
BSA, m^2^	1.5 (1.1, 1.8)	1.6 (1.3, 1.8)	1.6 (1.2, 1.8)	1.4 (1.0, 1.7)	1.5 (1.0, 1.8)	0.044*	0.4436	0.0021*	0.009*	0.0397*
SBP, mmHg	113(101,123)	117 (106, , 124)	114 (104, 123)	113 (102, 126)	111 (96, 122)	0.239	0.575	0.722	0.396	0.059
SBP, z-score	0.26 (− 0.62, 1.11)	0.50 (− 0.26, 1.20)	0.42 (− 0.56, 1.09)	0.44 (− 0.37, 1.37)	0.05 (− 0.90, 0.87)	0.287	0.016*	0.412	0.617	0.016*
Heart rate, bpm	82 (70, 93)	80 (68, 89)	78 (67, 89)	85 (73, 95)	82 (69, 93)	0.628	0.518	0.001*	0.171	0.6528
%MHR	41(36, 47)	40 (34, 46)	40 (35, 45)	43 (37, 48)	41(36, 47)	0.513	0.797	0.006*	0.174	0.564
QRS interval, ms	100 (90, 116)	84 (78, 92)	100 (90, 112)	106 (92, 120)	100 (88, 112)	< 0.001*	< 0.001*	0.002*	< 0.001*	< 0.001
Conventional CMR variables										
EDV_*i*_, mL/BSA (N = 467)	107 (92, 134)	82 (73, 93)	94 (79, 115)	115 (99, 142)	111 (95, 143)	< 0.001*	< 0.001*	< 0.001*	< 0.001*	< 0.001*
ESV_*i*_, mL/BSA (N = 467)	50 (39, 68)	32 (27, 38)	43 (32, 54)	56 (46, 75)	52 (39, 68)	< 0.001*	< 0.001*	< 0.001*	< 0.001*	< 0.001*
Mass_*i*_, grams/BSA (N = 449)	55 (46, 70)	48 (42, 54)	53 (46, 66)	56 (45, 70)	57 (47, 74)	< 0.001*	0.001*	0.839	0.001*	< 0.001*
EF % (N = 467)	54 (47, 58)	60 (56, 66)	55 (50, 60)	51 (45, 56)	54 (46, 60)	< 0.001*	< 0.001*	< 0.001*	< 0.001*	< 0.001*
Indexed stroke volume (N = 467)	57 (48, 66)	51 (44, 55)	51 (44, 59)	58 (52, 67)	61 (50, 72)	0.003*	0.502*	< 0.001*	< 0.001*	< 0.001*
Aortic flow, L/min (N = 359)	3.0 (2.5, 3.6)	3.6 (3.2, 3.8)	3.0 (2.4, 3.5)	3.2 (2.7, 3.9)	2.9 (2.4, 3.5)	0.002*	0.001*	0.014*	0.055	0.004*
GCS, % (N = 503)	− 14.6 (− 16.8, − 12.2)	− 17.9 (− 19.7, − 17.0)	− 16.6 (− 18.5, − 14.3)	− 13.6 (− 15.6, − 11.2)	− 14.0 (− 16.3, − 12.0)	< 0.001*	0.001*	< 0.001*	< 0.001*	< 0.001*
GRS, % (N = 503)	22.2 (17.66, 27.28)	29.40 (27.29, 33.63)	26.18 (21.71, 31.34)	19.78 (15.56, 24.57)	21.20 (17.28, 26.31)	< 0.001*	0.002*	< 0.001*	< 0.001*	< 0.001*

Values are medians (interquartile range) or counts (%)

*LV* left ventricle, *RV* right ventricle, *CMR* cardiac magnetic resonance, *BSA* body surface area, *SBP* systolic blood pressure, *%MHC* percent maximum heart rate, *EDV*_*i*_ indexed ventricular end-diastolic volume, *ESV*_*i*_ indexed ventricular end-systolic volume, *Mass*_*i*_ indexed ventricular mass, *EF* ejection fraction, *GCS* global circumferential strain, *GRS* global radial strain

*Indicates p-value < 0.05

**Table 2:** Dyssynchrony indices

	All Fontan patients (N = 503)	Comparison LV (N = 42)	Fontan subgroups			p-value				
			LV (N = 157)	RV (N = 190)	Mixed (N = 156)	All Fontan vs. comparison LV	LV vs. comparison LV	LV vs. RV	RV vs. comparison	Mixed vs. comparison LV
SDTTP-CS, ms	51 (40, 71)	45 (37, 51)	43 (34, 57)	53 (42, 71)	63 (45, 84)	0.011*	0.735	< 0.001*	0.023*	< 0.001*
SDTTP-RS, ms	50 (38, 70)	44(36, 53)	42(34, 56)	52 (41, 70)	57 (45, 80)	0.039*	0.460	< 0.001*	0.014*	< 0.001*
SDTTP-RSR, ms	96 (75, 122)	75 (55, 95)	81 (67, 102)	102 ‘982, 131)	102 (78, 132)	< 0.001*	0.214	< 0.001*	< 0.001*	< 0.001*
MOWD-CS, ms	56 (38, 81)	42 (32, 49)	49 (34, 73)	57 (38, 81)	60 (41, 88)	0.001*	0.028*	0.041*	0.001*	< 0.001*
MOWD-RSR, ms	84 (54, 130)	61 (40, 80)	72 (46, 107)	91(56, 144)	85 (57, 123)	< 0.001*	0.035*	0.004*	< 0.001*	< 0.001*
BAD1-CS, ms	38 (22, 65)	33 (21, 67)	34 (22, 55)	44 (22, 66)	38 (22, 72)	0.932	0.610	0.137	0.816	0.625
BAD1-CSR, ms	60 (23, 139)	30 (0, 57)	48 (24, 133)	63 (20, 144)	64 (25, 140)	0.003*	0.014*	0.535	0.008*	0.535
BAD2 CS, ms	29 (13, 47)	32 (15, 48)	23 (13, 36)	30 (13, 49)	35 (16, 54)	0.362	0.024*	0.029*	0.523	0.029*
BAD2-CSR, ms	50 (16, 96)	20 (13, 48)	36 (15, 75)	60 (17, 104)	55 (16, 112)	0.003*	0.045*	0.019*	0.001*	0.019*

Values are medians (interquartile range); *SDTTP* standard deviation time to peak, *CS* circumferential strain, *RS*, radial strain *RSR* radial strain rate, *MOWD* maximum opposing wall delay, *BAD1* base to apex delay for one slice, *BAD 2* base to apex delay for two slices

*Indicates p-value < 0.05

## Results

### Baseline characteristics

A total of 576 patients with a Fontan circulation and CMR were screened for eligibility and the final study cohort included 503 patients with a median age of 15.2 (IQR 10.3, 21.3) years and 42 comparison group patients with median age of 15.7 (IQR 11.0, 19.7) years. A total of 73 patients were excluded from after screening due to inadequate imaging. Common reasons for inadequate imaging were lack of full short-axis image stack (51 patients), inability to obtain reliable feature tracking due to motion artifact or blurring (10 patients), metallic artifact (9 patients), imaging performed at a lower than standard 30 phases/cardiac cycle temporal resolution (3 patients). Ventricular morphology was RV in 190 (38%), LV in 157 (31%), and mixed type in 156 (31%) of the patients with a Fontan circulation. The type of Fontan operations were lateral tunnel (69%), extracardiac conduit (20%), RA-PA (10%), and RA-RV (1%). Compared to the comparison group, Fontan patients had a lower BSA and were more frequently male (Table 1). Compared to the LV group, patients in the RV group were younger, had a lower BSA, and had a higher heart rate. The indications for CMR in the comparison group were family history of cardiomyopathy (N = 24, 57%), concern for arrhythmogenic right ventricular dysplasia (N = 3, 7%), family history of a bicuspid aortic valve (N = 3, 7%), family history of sudden cardiac death (N = 3, 7%), and concern for other abnormality on prior echocardiogram (N = 9, 21%). The patients undergoing evaluation for cardiomyopathy had a normal CMR as well as normal clinical and genetic evaluations (when available) at most recent follow-up.

### Relationship of dyssynchrony to ventricular morphology and conventional CMR metrics

**Fig. 3:**
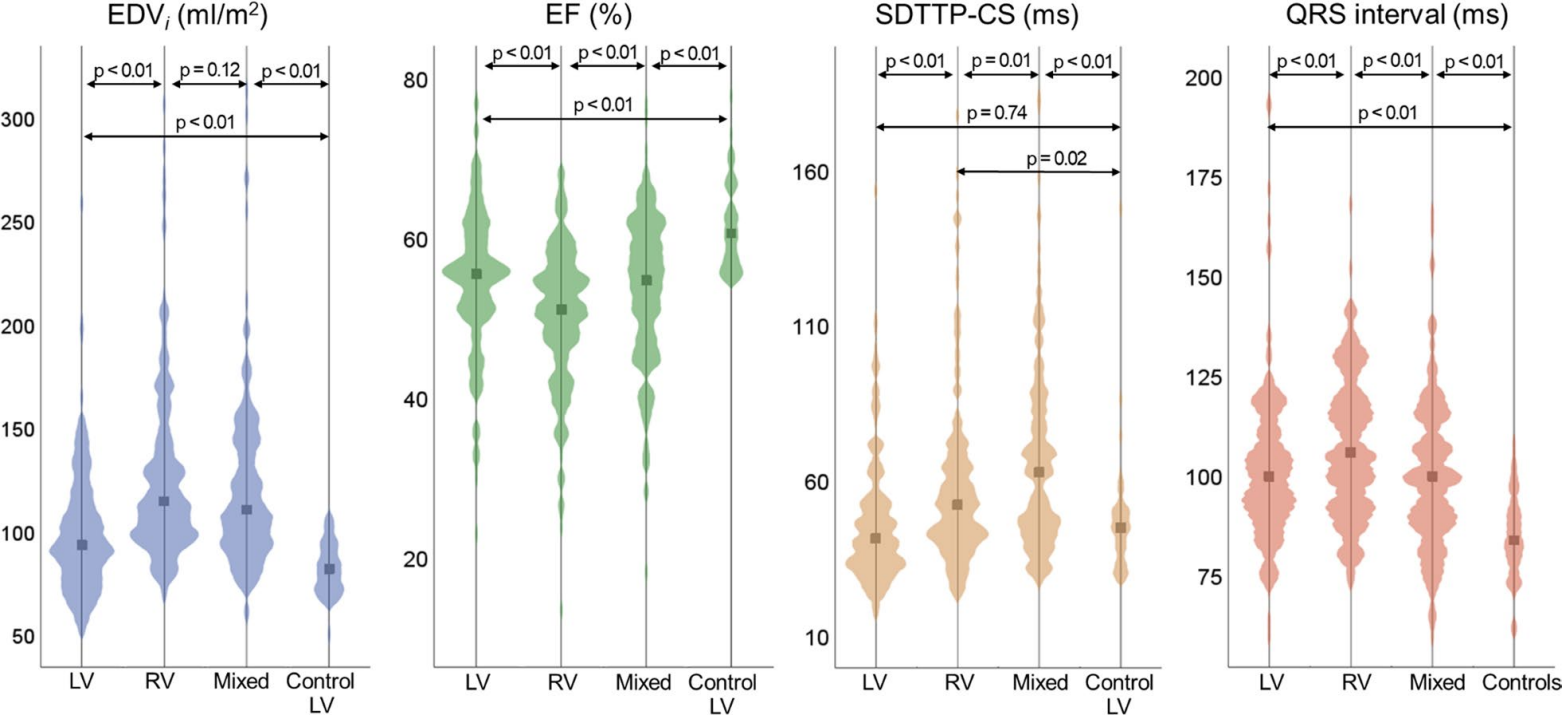
Violin plots comparing EDV_*i*_, EF, SDTTP-CS, and QRS interval between different ventricular morphologies and controls. *EDV*_*i*_ indexed ventricular end-diastolic volume, *EF* ejection fraction, *SDTTP-CS* standard deviation time to peak circumferential strain, *LV* left ventricle, *RV* right ventricle

**Fig. 4:**
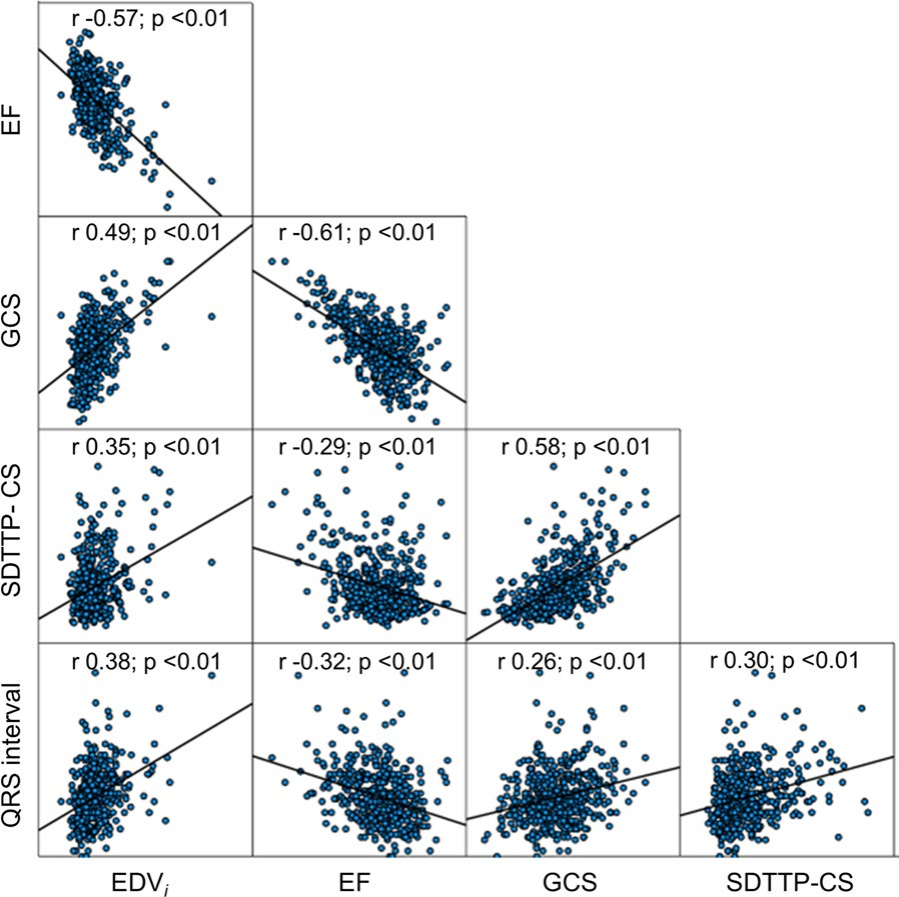
Scatterplot matrix showing correlation between ventricular size, function, dyssynchrony, and QRS duration for the Fontan cohort (N = 503). *EDV*_*i*_ indexed end diastolic volume, *EF* ejection fraction, *GCS* global circumferential strain, *SDTTP-CS* standard deviation time to peak circumferential strain

Demographic, ECG, CMR, and dyssynchrony parameters for the study cohort are presented in Tables 1 and 2. Median number of days between ECG and CMR was 0 (IQR 1, 4 ) days. In contrast to the comparison cohort, the Fontan patients had larger systemic ventricular volumes and mass, and lower EF, indexed stroke volume, GCS, GRS, and longer QRS interval. Details of all 24 CMR-derived dyssynchrony indices for the Fontan cohort and the comparison group are presented in Additional file 1: Table S1. Figure 3 shows the distributions for EDV_*i*_, EF, SDTTP-CS, and QRS interval across ventricular morphology types and comparison subjects. Within the Fontan cohort, the RV and mixed groups have higher volumes, lower EF, longer QRS duration, and higher SDTTP-CS. SDTTP-CS for the LV group was similar that of the comparison LV group. Ventricular size and function measurements demonstrated modest correlation with QRS duration and SDTTP-CS (Fig. 4; Additional file 1: Figure S1). Among all the evaluated dyssynchrony metrics, SDTTP-CS, SDTTP-RS, and SDTTP-RSR had the highest correlation with measures of ventricular function (GCS, GRS, and EF; correlation coefficients ranging from 0.3 to 0.6; Additional file 1: Table S2).

### Primary outcomes: death or heart transplantation

**Fig. 5:**
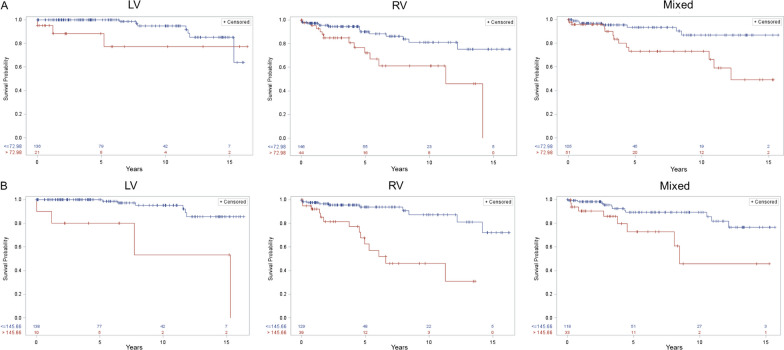
Kaplan–Meier plots depicting freedom from the composite outcome (death or transplant listing) stratified by presence of dyssynchrony (SDTTP-CS > 73 ms, **A**) and ventricular dilation (EDV_*i*_ > 146 ml/m^2^, **B**). **A**) Among patients with higher dyssyynchrony, patients with LV morphology experienced the greatest freedom from the composite outcome. The overall logrank p-value testing are for LV (p < 0.06), RV (p < 0.002) and mixed (p = 0.005). **B**) The overall logrank p-value testing are for LV (p < 0.001), RV (p < 0.001) and mixed (p = 0.006). The pairwise adjusted p-values using Tukey–Kramer for multiple comparisons remained significant for all tests

With a median follow-up period of 4.3 (IQR 1.3, 7.8) years, 57 (11.3%) of the patients met the composite primary outcome with 46 deaths, 8 heart transplantations, and 18 heart transplant listings. In the RV group, 28 (14.7%) patients had the outcome compared to 19 (12.2%) in the mixed group, and 10 (6.4%) in the LV group. On bivariate Cox regression analysis, D/HTx was associated with RV or mixed ventricular morphology, lower blood pressure, higher heart rate, longer QRS, ventricular dilation, lower EF, and higher dyssynchrony indices (Table 3). A multivariable logistic regression model found that RV morphology (HR ratio 2.3; 95% CI 1.1, 4.9; p-value 0.026), EDV_*i*_ per 10 ml (HR 1.1; 95% CI 1.1, 1.2; p-value < 0.001) and percent MHR (HR ratio 1.5; 95% CI 1.2, 2.0; p-value 0.003) were independently associated with D/HTx, however, dyssynchrony metrics were not on multivariable analyses (Table 4). Figure 5 depicts Kaplan Meier plots for each ventricular morphology type stratified by cut off values for EDV_*i*_ and SDTTP-CS. The estimated 3-year percent D/HTx was 7.9% (95% CI 3.7, 12.0) for single RVs, 3.5% (95% CI 0.4, 6.4) for mixed typed ventricles, and 1.4% (95% CI 0.0, 3.2) for single LVs (p-value = 0.005). Using the cut off point of 73 ms for SDTTP-GCS that maximizes the log rank statistic, those with a SDTTP-CS of  > 73 ms had a higher Kaplan Meier estimate of 3-year percent D/HTx (12.5%; 95% CI 3.4, 15.5) compared to those below the cut off (3.3%; 95% CI 1.3, 5.1). Patients with EDV_*i*_ > 146 ml/m^2^ had a higher rate of the primary outcome than those with EDV_*i*_ ≤ 146 ml/m^2^ (17% [95% CI 6.4, 23.11) vs. 2.8% [95% CI 0.95, 4.6]; p-value 0.002).

**Table 3:** Bivariate Cox regression analysis for time to death or heart transplant listing

Predictor	Death or heart transplantation or listing (N = 57); HR (95% CI)	p-value
Age at CMR (years)	0.9 (0.6, 1.1)	0.280
Sex (male)	1.0 (0.6, 1.7)	0.950
Heart rate (per 10 bpm)	1.3 (1.2, 1.5)	< 0.001*
%MHR (per 10 units)	1.7 (1.3, 2.2)	< 0.001*
SBP, mmHg (per 10 units)	0.8 (0.7, 1.0)	0.030*
RA-PA or RA-RV Fontan (ref: extracardiac Fontan)	0.6 (0.2, 1.4)	0.227
Lateral tunnel Fontan (ref: extracardiac Fontan)	0.6 (0.3, 1.2)	0.123
RV morphology (ref: LV)	3.2 (1.5, 6.6)	0.002*
Mixed ventricular morphology (ref: LV)	2.4 (1.1, 5.3)	0.023*
QRS interval, ms (per 10 units)	1.1 (1.0, 1.3)	0.025*
EDV_*i*_, ml/m^2^ (per 10 units)	1.2 (1.1, 1.2)	< 0.001*
ESV_*i*_, ml/m^2^ (per 10 units)	1.2 (1.1, 1.2)	< 0.001*
Mass_*i*_, gm/m^2^ (per 10 units)	1.2 (1.1, 1.3)	< 0.001*
EF, % (per 10 units)	0.6 (0.5, 0.8)	< 0.001*
GCS, % (per 10 units)	7.5 (3.5, 15.8)	< 0.001*
GRS, % (per 10 units)	0.4 (0.3, 0.6)	< 0.001*
SDTTP-CS, ms (per 10 units)	1.2 (1.1, 1.3)	0.022*
SDTTP-RS, ms (per 10 units)	1.2 (1.1, 1.3)	< 0.001*
SDTTP-RSR, ms (per 10 units)	1.1 (1.0, 1.2)	< 0.001*
MOWD-RSR, ms (per 10 units)	1.0 (0.99, 1.1)	0.118
MOWD-CS, ms (per 10 units)	1.0 (0.97, 1.1)	0.316
BAD1-CS, ms (per 10 units)	1.0 (0.9, 1.1)	0.729
BAD2 CS, ms (per 10 units)	1.0 (0.9, 1.1)	0.468
BAD1-CSR, ms (per 10 units)	1.0 (0.97, 1.0)	0.930
BAD2-CSR, ms (per 10 units)	1.0 (0.96, 1.0)	0.873

*CMR* cardiac magnetic resonance, *%MHR* percent maximal heart rate, *SBP* systolic blood pressure, *RV* right ventricle, *LV* left ventricle, *EDV*_*i*_ indexed ventricular end-diastolic volume, *ESV*_*i*_ indexed ventricular end-systolic volume, *Mass*_*i*_ indexed ventricular mass, *EF* ejection fraction, *GCS* global circumferential strain, *SDTTP* standard deviation time to peak, *GCS* global circumferential strain, *GRS* global radial strain, *SDTTP* standard deviation time to peak, *CS* circumferential strain, *RS* radial strain, *RSR* radial strain rate, *MOWD* maximum opposing wall delay, *BAD1* base to apex delay for one slice, *BAD 2* base to apex delay for two slices

*Indicates p-value < 0.05

**Table 4:** Multivariate Cox regression model for time to death or heart transplant listing

Predictor	aHR (95% CI)	p-value
RV morphology (ref: LV morphology)	2.32 (1.11, 4.88)	0.026*
Mixed ventricular morphology (ref: LV morphology)	1.63 (0.74, 3.58)	0.223
EDV_*i*_, ml/m^2^ (per 10 units)	1.14 (1.09, 1.19)	< 0.001*
Percent maximum heart rate, 10% (per 10 units)	1.51 (1.15, 1.99)	0.003*

Multiple imputation used for missing data. (N = 503; 57 outcomes; model c-statistic 0.79)

*aHR* adjusted hazard ratio, *RV* right ventricle, *LV* left ventricle, *EDV*_*i*_ indexed ventricular end-diastolic volume

*Indicates p-value < 0.05

## Discussion

This retrospective analysis of a large cohort of patients with a Fontan circulation provides a comprehensive characterization and analysis of mechanical dyssynchrony in single ventricles using CMR-FT. The cohort covers a wide breadth of single ventricular morphology, including dominant right, left, and mixed type ventricles. Our data demonstrate that ventricles in the Fontan circulation have a higher degree of dyssynchrony compared to control LVs and that dyssynchrony is associated with reduced ventricular function and D/HTx. RV and mixed morphology subtypes had a higher degree of dyssynchrony compared to those with LV morphology. Conversely, in those with LV morphology, dyssynchrony indices were more similar to those in the comparison group LVs. These results contribute to the growing body of literature demonstrating adverse ventricular remodeling in the Fontan circulation and highlight new risk factors for increased morbidity and mortality, which are critical to understanding and managing a growing population surviving into adulthood.

Data regarding the characterization and patterns of dyssynchrony in patients with a Fontan circulation are growing but remain to be fully elucidated. Both adult and pediatric patients with heart failure are known to have more dyssynchronous patterns of electrical and mechanical ventricular contraction, which is associated with worse LV and RV systolic function and increased morbidity [25, 26]. Similar to adults, no single gold standard measurement of mechanical dyssynchrony has gained universal acceptance or validation in pediatrics [27, 28]. Prior studies have assessed dyssynchrony in the single ventricle population using mid-ventricular or apical slices, typically using a 6-segment model [15, 29, 30]. The majority of these studies are echocardiogram-based and are limited by incomplete visualization of the entire myocardium This study builds on prior work by capturing the performance of the entire ventricle by analyzing all segments of myocardium to estimate the SDTTP rather than estimate dyssynchrony metrics on a portion or single slice of the myocardium. Although not associated with the outcome, the study also explores new methods of MOWD and BAD to characterize dyssynchrony. Our metrics of were only modestly correlated with EF, perhaps because ventricles in the Fontan circulation have increased sphericity compared to the normal bullet shaped left ventricles. While gathering normative CMR data from healthy controls remains a challenge, the inclusion of 42 age-matched comparison group patients in this study sheds some light on normal dyssynchrony indices. No normative data exist for dyssynchrony measures by ventricular morphology, and there is no accepted threshold for when measurements become clinically significant. A prior study analyzed dyssynchrony in 100 patients with a Fontan circulation using long-axis views and reported that those with a SDTTP longitudinal strain of > 63.5 ms was associated with a composite outcome of heart failure, unplanned hospitalization, or death [15]. These findings are similar to the SDTTP-CS cut-off of 73 ms for D/HTx that we found in the present study.

In biventricular hearts with acquired heart diseases, QRS duration correlates with markers of ventricular dyssynchrony, dilation, and heart failure [31, 32]. QRS duration has also been identified as a prognostic indicator in CHD, such as tetralogy of Fallot; although, recent studies have shown that its effect may not be independent of LV dysfunction [33]. Similar data in the Fontan population are limited. In small echocardiography-based studies on pediatric patients with a Fontan circulation, longer QRS duration has been associated with increased incidences of dyssynchrony [34] with worse systolic function. Additionally, catheterization-based studies in this population have shown QRS prolongation is linked to poor hemodynamics with higher filling pressures and lower cardiac index [35, 36]. The current study analyzes a relatively large cohort and demonstrates associations between QRS duration, ventricular dyssynchrony, and adverse clinical outcomes. Unlike previously identified unmodifiable risk factors, the contribution of CMR-based dyssynchrony metrics with poor outcomes can be informative in the management of patients with CHD. In patients with and without CHD, cardiac resynchronization therapy (CRT) has become a treatment strategy for individuals with heart failure and ventricular dyssynchrony [37, 38]. In single ventricle patients, the best approach to resynchronization is unknown and needs to be considered on a case-by-case basis**.** The discovery of CMR markers of mechanical dyssynchrony may offer an opportunity to identify high-risk Fontan patients who may benefit from CRT and subsequently follow their response to CRT. Furthermore, segmental analysis using FT to compare the timing and peak offset for GCS may inform lead placement to achieve optimal synchronous contraction in an individual patient.

This study reaffirmed that impaired ventricular EF, ventricular dilation, and RV or mixed ventricular morphology are established risk factors for poor clinical outcomes in patients with a Fontan circulation [3, 39, 40]. Multivariable analysis revealed that the association of adverse outcomes with markers of dyssynchrony is not independent of established conventional markers such as ventricular systolic dysfunction and dilation. This is in contrast to recently published findings by Schafer et al. who found that dyssynchrony metrics derived from analyzing the global strain time curve morphology using principal component analysis were independent predictors of protein losing enteropathy, plastic bronchitis, transplant, and death. It may be that their novel approach is more sensitive to capture dyssynchrony, however, it is interesting that in their cohort ventricular dilation was only an independent predictor of poor outcomes in patients with a single RV [41]. Our findings of lower event-free survival in RV or mixed ventricular subtypes agree with prior large multicenter observational studies on Fontan patients, which have demonstrated lower long-term survival in these groups [3]. On stratified survival analysis, however, dyssynchronous single RVs had worse outcomes compared to dyssynchronous single LVs and mixed type ventricles. This suggests that ventricular dominance remains a valuable predictor of outcomes, even amongst patients with failing Fontan physiology. Furthermore, higher heart rates were independently associated with D/HTx. Perhaps the orientation of myofibers, myocardial fibrosis, and the lack of ventricular–ventricular interactions in the non-LV Fontans contribute to the development of dyssynchronous myocardial contraction, heart failure [42–44].

## Limitations

This study has several limitations that must be acknowledged. The generalizability of the findings to the Fontan population as a whole may be limited by a single-center retrospective design that uses availability of a CMR as an inclusion criterion. The study focused on mechanical performance of the ventricles and did not analyze markers of fibrosis such as late gadolinium enhancement or myocardial T1 measurements, as that would have resulted in a substantially smaller cohort. The relatively low temporal resolution of CMR-FT analysis must also be acknowledged. Our cohort has a selection bias as pediatric patients referred for a CMR tend to be older and those with pacemakers and defibrillators were excluded. Moreover, a referral bias for CMR testing may lead to sicker and more symptomatic patients being overrepresented in the cohort. Lastly, our cohort had a relatively small number of age-matched controls for comparison due to availability.

## Conclusions

Compared to control LVs, single right and mixed morphology ventricles in the Fontan circulation exhibit a higher degree of mechanical dyssynchrony as evaluated by CMR-FT. Dyssynchrony indices correlate with ventricular size and function and are associated with death or need for heart transplantation. These data add to the growing understanding of progressive decline in ventricular performance in the Fontan population.

### Supplementary Information

**Additional file 1: Table S1.** Dyssynchrony metrics for comparisons and patients by ventricular morphology. **Table S2.** Correlation between dyssynchrony metrics and measures of systolic function. **Figure S1.** Scatterplot matrix showing correlation between ventricular size, function, dyssynchrony, and QRS duration for the Fontan cohort and separated by ventricular morphology

## Data Availability

Data and materials are available upon request.

## Abbreviations

AIC: Akaike information criterion
BAD: Base-to-apex delay
CMR: Cardiac magnetic resonance
CO_*i*_: Indexed cardiac output
CS: Circumferential strain
D/HTx: Death or heart transplant listing
ECG: Electrocardiogram
EDV: End-diastolic volume
EDV_*i*_: Indexed end-diastolic volume
EF: Ejection fraction
ESV_*i*_: Indexed end-systolic volume
FT: Feature tracking
GCD: Global circumferential displacement
GCS: Global circumferential strain
GCSR: Global circumferential strain rate
GRD: Global radial displacement
GRS: Global radial strain
GRSR: Global radial strain rate
IQR: Interquartile ranges
LV: Left ventricle
Mass_i_: Indexed ventricular mass
MHR: Maximum heart rate
MOWD: Maximum opposing wall delay
RS: Radial strain
RV: Right ventricle
SDTTP: Standard deviation of time-to-peak
SV_*i*_: Indexed stroke volume
