# Supplementary material for: Ventricular dyssynchrony late after the Fontan operation is associated with decreased survival
Source: J Cardiovasc Magn Reson. 2023 Nov 20;25:66. doi: 10.1186/s12968-023-00984-3 (PMC10658858; doi:10.1186/s12968-023-00984-3)

**Table S1.** Dyssynchrony metrics for comparisons and patients by ventricular morphology

|  |  |  | **Fontan Subgroups** | | | **p-value** | | | | |
| --- | --- | --- | --- | --- | --- | --- | --- | --- | --- | --- |
|  | All Fontan Patients (N=503) | Comparison group LV (N=42) | LV (N=157) | RV (N=190) | Mixed (N=156) | All Fontan vs. comparison group LV | LV vs. comparison group LV | LV vs. RV | RV vs. comparison group | Mixed vs. comparison group LV |
| SDTTP-RSR, ms | 96 (75-122) | 75 (55-95) | 81 (67-102) | 102 ‘982-131) | 102 (78-132) | <0.001* | 0.214 | <0.001* | <0.001* | <0.001* |
| SDTTP-RS, ms | 50 (38-70) | 44(36-53) | 42(34-56) | 52 (41-70) | 57 (45-80) | 0.039* | 0.460 | <0.001* | 0.014* | <0.001* |
| SDTTP-RD | 61 (48-76) | 49 (39-60) | 57 (44-68) | 63 (49-76) | 62 (48-79 | <0.001* | 0.021* | 0.008* | <0.001* | 0.004* |
| SDTTP-CSR | 115 (86-150) | 83 (59-115) | 94 (77-128) | 124 (91-162) | 123 (93-155) | <0.001* | 0.022* | <0.001* | <0.001* | <0.001* |
| SDTTP-CS, ms | 51 (40-71) | 45 (37-51) | 43 (34-57) | 53 (42-71) | 63 (45-84) | 0.011* | 0.735 | <0.001* | 0.023* | <0.001* |
| SDTTP-CD | 107 (90-126) | 119 (106-134) | 111 (95-132) | 106 (90-120) | 106 (88-125) | <0.001* | 0.025* | 0.022* | <0.001* | 0.058 |
| MOWD-RS | 55 (35-78) | 39 (28-49) | 48 (34-68) | 58 (36-80) | 58 (36-82) | <0.001* | 0.010* | 0.031* | <0.001* | <0.029* |
| MOWD-RD | 69 (46-100) | 59 (40-79) | 71 (51-104) | 69 (45-97) | 68 (41-99) | 0.076 | 0.024* | 0.278 | 0.130 | 0.116 |
| MOWD-CSR | 104 (71-151) | 61 (43-92) | 95 (61-144) | 122 (76-178) | 101 (70-142) | <0.001* | <0.001* | <0.001* | <0.001* | 0.292 |
| MOWD-CS, ms | 56 (38-81) | 42 (32, 49) | 49 (34-73) | 57 (38-81) | 60 (41-88) | 0.001* | 0.028* | 0.041* | 0.001* | <0.001* |
| MOWD-CD | 98 (67-128) | 95 (69-135) | 98 (67-130) | 95 (68-126) | 99 (65-130) | 0.909 | 0.967 | 0.605 | 0.758 | 0.949 |
| MOWD-RSR, ms | 84 (54-130) | 61 (40-80) | 72 (46-107) | 91(56-144) | 85 (57-123) | <0.001* | 0.035* | 0.004* | <0.001* | <0.001* |
| BAD2-RSR | 29 (13-65) | 20 (0.38-38) | 29 (14-56) | 31 (13-75) | 28 (13-62) | 0.049* | 0.073 | 0.456 | 0.035* | 0.763 |
| BAD2-RS | 27 (13-44) | 29 (13-47) | 23 (12-37) | 28 (11-44) | 32 (14-53) | 0.568 | 0.179 | 0.131 | 0.635 | 0.009* |
| BAD2-RD | 33 (14-56) | 25 (15-33) | 28 (14-50) | 34 (12-59) | 35 (16-58) | 0.102 | 00.418 | 0.242 | 0.090 | 0.076 |
| BAD2-CSR, ms | 50 (16-96) | 20 (13-48) | 36 (15-75) | 60 (17-104) | 55 (16-112) | 0.003* | 0.045* | 0.019* | 0.001* | 0.019* |
| BAD2 CS, ms | 29 (13-47) | 32 (15-48) | 23 (13-36) | 30 (13-49) | 35 (16-54) | 0.362 | 0.024* | 0.029* | 0.523 | 0.029* |
| BAD2-CD | 68 (33-115) | 100 (28-152) | 68 (36-117) | 68 (34-113) | 68 (28-118) | 0.076 | 0.108 | 0.766 | 0.066 | 0.974 |
| BAD1-RSR | 38 (19-85) | 31 (0-67) | 43 (24-83 | 34 (16-88) | 37 (19-92) | 0.150 | 0.130 | 0.355 | 0.295 | 0.943 |
| BAD1-RS | 36 (21-63) | 29 (0-70) | 32 (19-58) | 40 (21-64) | 39 (20-67) | 0.921 | 0.729 | 0.213 | 0.873 | 0.114 |
| BAD1-RD | 46 (23-71) | 29 (16-53) | 47 (22-69) | 50 (23-73) | 44 (25-70) | 0.048* | 0.122 | 0.424 | 0.040* | 0.823 |
| BAD1-CSR, ms | 60 (23-139) | 30 (0-57) | 48 (24-133) | 63 (20-144) | 64 (25-140) | 0.003* | 0.014* | 0.535 | 0.008* | 0.535 |
| BAD1-CS, ms | 38 (22-65) | 33 (21, 67) | 34 (22-55) | 44 (22-66) | 38 (22-72) | 0.932 | 0.610 | 0.137 | 0.816 | 0.625 |
| BAD1-CD | 93 (42-169) | 115 (48-188) | 97 (35-175) | 93 (44-174) | 91 (44-168) | 0.353 | 0.380 | 0.929 | 0.376 | 0.850 |

**Abbreviations:** SDTTP, standard deviation time to peak; RSR radial strain rate; RS, radial strain; RD, radial displacement; CSR, circumferential strain rate; CR, circumferential strain; CD, circumferential displacement; MOWD, maximum opposing wall; BAD2-RSR, base to apex delay of 2 most apical and basal slices; BAD1, base to apex delay of the single apical and basal most slice.

**Table S2.** Correlation between dyssynchrony metrics and measures of systolic function

|  | GCS | GRS | EF |
| --- | --- | --- | --- |
| GCS | 1 | -.984** | -.613** |
| GRS | -.984** | 1 | .588** |
| EF | -.613** | .588** | 1 |
| SDTTP-CS | .577** | -.503** | -.293** |
| SDTTP-RS | .577** | -.505** | -.296** |
| SDTTP-RSR | .499** | -.465** | -.409** |
| SDTTP-RD | .394** | -.371** | -.202** |
| MOWD-RS | .367** | -.321** | -.128** |
| MOWD-CS | .366** | -.322** | -.104* |
| MOWD-RSR | .340** | -.316** | -.287** |
| SDTTP-CSR | .304** | -.276** | -.260** |
| MOWD-RD | .236** | -.219** | -.160** |
| BAD2-RD | .234** | -.250** | -.151** |
| BAD2-RSR | .227** | -.199** | -.150** |
| MOWD-CSR | .224** | -.212** | -.192** |
| BAD2-CS | .200** | -.185** | -.111* |
| BAD2-RS | .200** | -.184** | -.111* |
| BAD1-RD | .157** | -.175** | -.114* |
| BAD1-RSR | .132** | -.118** | -0.074 |
| BAD2-CSR | .130** | -.108* | -0.069 |
| BAD1-CS | .129** | -.114* | -0.054 |
| BAD1-RS | .108* | -.095* | -0.026 |
| BAD1-CSR | 0.081 | -0.067 | -0.036 |
| BAD1-CD | 0.021 | -0.033 | -0.004 |
| SDTTP-CD | 0.006 | -0.021 | -0.007 |
| MOWD-CD | -0.005 | 0.001 | 0.015 |
| BAD2-CD | -0.045 | 0.040 | 0.055 |

Numbers represent Pearson’s correlation coefficients. Variables are arranged in the descending order of the correlations coefficient in the GCS column. **p-value <0.01, *p-value <0.05.

**Abbreviations:** GCS, global circumferential strain; GRS, global radial strain; EF, ejection fraction; SDTTP, standard deviation time to peak; RSR radial strain rate; RS, radial strain; RD, radial displacement; CSR, circumferential strain rate; CR, circumferential strain; CD, circumferential displacement; MOWD, maximum opposing wall; BAD2-RSR, base to apex delay of 2 most apical and basal slices; BAD1, base to apex delay of the single apical and basal most slice.

Figure S1. Scatterplot matrix showing correlation between ventricular size, function, dyssynchrony, and QRS duration for the Fontan cohort and separated by ventricular morphology


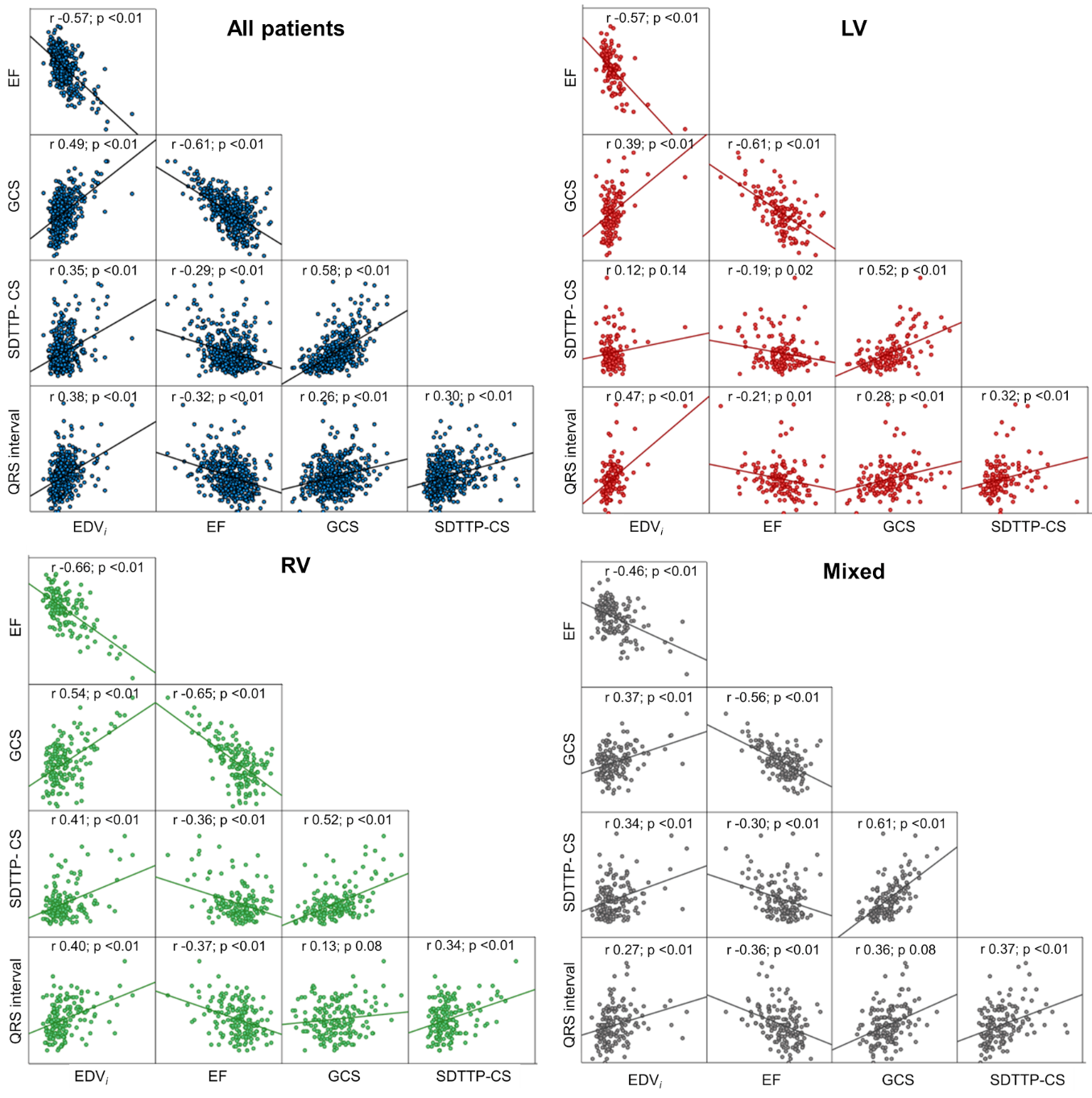

Supplement: Supplementary file 1 — Additional file 1: Table S1. Dyssynchrony metrics for comparisons and patients by ventricular morphology. Table S2. Correlation between dyssynchrony metrics and measures of systolic function. Figure S1. Scatterplot matrix showing correlation between ventricular size, function, dyssynchrony, and QRS duration for the Fontan cohort and separated by ventricular morphology [file 12968_2023_984_MOESM1_ESM.docx]
